# Safety and Pharmacokinetics of Glecaprevir/Pibrentasvir in Adults With Chronic Genotype 1–6 Hepatitis C Virus Infections and Compensated Liver Disease

**DOI:** 10.1093/cid/ciz022

**Published:** 2019-03-28

**Authors:** Edward Gane, Fred Poordad, Neddie Zadeikis, Joaquin Valdes, Chih-Wei Lin, Wei Liu, Armen Asatryan, Stanley Wang, Catherine Stedman, Susan Greenbloom, Tuan Nguyen, Magdy Elkhashab, Marcus-Alexander Wörns, Albert Tran, Jean-Pierre Mulkay, Carolyn Setze, Yao Yu, Tami Pilot-Matias, Ariel Porcalla, Federico J Mensa

**Affiliations:** 1 Auckland Clinical Studies, New Zealand; 2 The Texas Liver Institute, University of Texas Health Science Center, San Antonio; 3 AbbVie Inc., North Chicago, Illinois; 4 Christchurch Hospital and University of Otago, New Zealand; 5 Toronto Digestive Disease Associates, Ontario, Canada; 6 Research and Education, Inc, San Diego, California; 7 Toronto Liver Centre, Ontario, Canada; 8 Universitätsmedizin der Johannes-Gutenberg Universität, Mainz, Germany; 9 University Hospital of Nice, Digestive Centre, France; 10 Hôpital Centre Hospitalier Universitaire Saint-Pierre, Brussels, Belgium

**Keywords:** glecaprevir/pibrentasvir, HCV, compensated cirrhosis, chronic kidney disease, adverse event

## Abstract

**Background:**

Untreated, chronic hepatitis C virus (HCV) infection may lead to progressive liver damage, which can be mitigated by successful treatment. This integrated analysis reports the safety, efficacy, and pharmacokinetics (PK) of the ribavirin-free, direct-acting, antiviral, fixed-dose combination of glecaprevir/pibrentasvir (G/P) in patients with chronic HCV genotype 1–6 infections and compensated liver disease, including patients with chronic kidney disease stages 4 or 5 (CKD 4/5).

**Methods:**

Data from 9 Phase II and III clinical trials, assessing the efficacy and safety of G/P treatment for 8–16 weeks, were included. The presence of cirrhosis was determined at screening using a liver biopsy, transient elastography, or serum biomarkers. The objectives were to evaluate safety, the rate of sustained virologic response at post-treatment week 12 (SVR_12_), and steady-state PK by cirrhosis status.

**Results:**

Among 2369 patients, 308 (13%) were Child-Pugh Class A, including 20 with CKD 4/5. Overall, <1% of patients experienced an adverse event (AE) that led to G/P discontinuation or G/P-related serious AEs (SAEs). The most common AEs were headache and fatigue, occurring at similar frequencies with and without cirrhosis. SAEs were more common in patients with CKD 4/5, but all were unrelated to G/P. There were no cases of drug-induced liver injury or clinically relevant hepatic decompensation. SVR_12_ rates were 96.4% (297/308) with compensated cirrhosis and 97.5% (2010/2061) without cirrhosis. PK analysis demonstrated a 2.2-fold increase in glecaprevir exposure, but not pibrentasvir exposure, in patients with compensated cirrhosis.

**Conclusions:**

G/P was safe and efficacious in patients with compensated liver disease, including those with CKD 4/5.

**Clinical Trials Registration:**

NCT02243280, NCT02243293, NCT02604017, NCT02640482, NCT02640157, NCT02636595, NCT02642432, NCT02651194, and NCT02446717

## Teaser

Glecaprevir/pibrentasvir is a safe and efficacious pangenotypic, direct-acting antiviral regimen for hepatitis C virus patients with Child-Pugh A cirrhosis (including with chronic kidney disease stages 4 or 5), despite a ~2-fold increase in glecaprevir exposures in patients with compensated cirrhosis.

**(See the Editorial Commentary by Kiser on pages 1665–6.)**

Chronic hepatitis C virus (HCV) infection is characterized by the gradual development of hepatic fibrosis, progressing to cirrhosis in approximately 20–30% of patients. Without treatment, patients with compensated cirrhosis are at an approximately 6.4% risk of progressing to decompensated cirrhosis per annum and at increased risk for hepatocellular carcinoma (HCC) and all-cause mortality [1, 2]. However, achievement of sustained virologic response at post-treatment week 12 (SVR_12_) is associated with decreased risks of disease progression [3, 4]. Previous HCV treatment guidelines prioritized the treatment of patients with compensated cirrhosis, based on these potential benefits [5].

Direct-acting antiviral (DAA) regimen containing first-generation protease inhibitors, such as simeprevir, paritaprevir, and asunaprevir, were associated with safety concerns in patients with cirrhosis. Elevations in alanine aminotransferase (ALT) were associated with some of these first-generation protease inhibitors [6]. Next-generation protease inhibitors, like grazoprevir, glecaprevir, and voxilaprevir, exhibit more favorable safety profiles in patients with compensated cirrhosis, including low (<2%) rates of clinically relevant ALT elevations [7–9]. In patients with advanced cirrhosis, particularly those with decompensated cirrhosis, increased exposure to protease inhibitors was associated with events consistent with hepatic decompensation or drug-induced liver injury and, thus, are contraindicated in patients with decompensated cirrhosis [6].

The next-generation, DAA, fixed-dose combination of glecaprevir (nonstructural protein 3/4A [NS3/4A] protease inhibitor identified by AbbVie and Enanta) and pibrentasvir (nonstructural protein 5A [NS5A] inhibitor) is a pangenotypic, ribavirin (RBV)-free regimen for patients with compensated liver disease, with or without chronic kidney disease (CKD) stages 4 or 5, including those on dialysis [5]. Glecaprevir and pibrentasvir are minimally metabolized, primarily undergoing biliary-fecal excretion, with <1% excreted by the kidneys [10, 11]. In an integrated analysis of patients without cirrhosis, glecaprevir/pibrentasvir (G/P) demonstrated an overall SVR_12_ rate >97% and a favorable safety profile [12]. A dedicated Phase III study (EXPEDITION-1; N = 146) demonstrated a similar efficacy and safety profile in patients with compensated cirrhosis [8]. However, to date, no comprehensive, integrated analysis has reported the safety, efficacy, and pharmacokinetics of G/P in all patients with compensated cirrhosis from the clinical development program.

Here, we present an integrated safety, efficacy, and pharmacokinetic analysis of G/P across Phase II and III clinical trials in patients with compensated cirrhosis (n = 308) or patients without cirrhosis (n = 2061), in order to inform the risk-benefit profile of G/P treatment in patients with compensated liver disease, including those with CKD stages 4 or 5.

## METHODS

### Analysis Set

Data were pooled from patients receiving G/P in 9 Phase II and III clinical trials, wherein the regimen’s safety, efficacy, and pharmacokinetics were assessed in patients with compensated liver disease (SURVEYOR-I and -II; MAGELLAN-1; ENDURANCE-1, -2, -3, and -4; and EXPEDITION-1 and -4) [8, 13–19]. The integrated analysis set included all patients in the registrational program who received at least 1 dose of glecaprevir 300 mg and pibrentasvir 120 mg, either as separate tablets (Phase II formulation) or coformulated tablets dosed orally as 3 pills, for a total 300 mg/120 mg dose (Phase III formulation). Both formulations were taken once daily with food for 8, 12, or 16 weeks. Patients were assigned to treatment duration based on genotype, cirrhosis status, and treatment experience, as outlined in Supplementary Figure 1. All authors had access to the study data, and reviewed and approved the final manuscript for submission.

### Patients

Complete inclusion and exclusion criteria are reported in the Supplementary Materials. Adults (≥18 years of age) with chronic HCV who were positive for anti-HCV antibodies with a plasma HCV RNA viral load ≥10 000 IU/mL in Phase II or ≥1000 IU/mL in Phase III at the screening visit were eligible for enrollment. Patients with compensated cirrhosis, who met all other inclusion criteria, were eligible for enrollment in SURVEYOR-II, MAGELLAN-I, and EXPEDITION-1 and -4. The presence or absence of cirrhosis was assessed consistently across all the studies, using a hierarchical approach that included a liver biopsy, transient elastography (FibroScan), or screening Fibrotest and an aspartate aminotransferase–to-platelet ratio index, as outlined in the Supplementary Materials. All studies excluded patients with decompensated liver disease, HCC, or a hepatitis B virus coinfection at screening. Additional exclusion criteria, related to other laboratory parameters, are outlined in the Supplementary Materials.

Patients were included if they were either HCV treatment-naive or had prior treatment experience with interferon (IFN)/pegylated IFN ± RBV or sofosbuvir + RBV ± pegylated IFN. Prior treatment failures with NS5A inhibitors and/or NS3/4A protease inhibitors were only included in MAGELLAN-1. Human immunodeficiency virus–1/HCV coinfected patients were only allowed to enroll in the HCV genotype 1 clinical trial, ENDURANCE-1. Patients were included only if their screening creatinine clearance was ≥50 mL/min, except in the Phase III trial (EXPEDITION-4), dedicated for the evaluation of patients with stage 4 or 5 CKD (creatinine clearance <30 mL/min). All patients provided written informed consent. Clinical trials were designed and conducted in accordance with the Good Clinical Practice guidelines, the Declaration of Helsinki, and applicable local regulations, and with approval from independent ethics committees or institutional review boards at all study sites.

### Procedures

The HCV genotype was determined using the Versant HCV Genotype Inno LiPA Assay, Version 2.0 or higher (Siemens Healthcare Diagnostics, Tarrytown, NY) and confirmed by the phylogenetic analysis of viral sequences. Plasma HCV RNA was quantified by real-time reverse-transcriptase polymerase chain reaction for assessing the baseline viral load and SVR_12_; assay details are described in the Supplementary Materials. Safety was evaluated by monitoring the following: adverse events (AEs), vital signs, physical examination findings, electrocardiographies, and clinical laboratory tests. Patients were monitored for AEs throughout G/P treatment and until 30 days post-treatment for nonserious and serious AEs and up to 24 weeks post-treatment for all spontaneously reported, serious AEs. Treatment-emergent AEs were defined as any AE with an onset date after the first G/P dose and no more than 30 days after the last G/P dose. All AEs were coded using the Medical Dictionary for Regulatory Activities. Study investigators assessed each AE for its possible relationship to the study drugs. Per the study protocol, all enrolled patients in Phase III studies were to collect blood samples at each study visit during the treatment period, for the pharmacokinetic assessment of glecaprevir and pibrentasvir concentrations. Patients who consented to participate in optional intensive pharmacokinetic sampling would have additional blood samples collected on Study Day 1 and Week 4, up to 6 hours postdose, during the treatment period. Glecaprevir and pibrentasvir plasma concentrations were measured by AbbVie using a validated assay method [20].

### Objectives

The primary objective of this integrated analysis was to determine the safety of G/P by evaluating the characteristics of reported AEs and the number and percentage of G/P-treated patients who reported treatment-emergent AEs and laboratory abnormalities, both in total and stratified by cirrhosis status and the presence or absence of CKD stages 4 or 5. Additional objectives included evaluating the sustained virologic response (HCV RNA < lower limit of quantification) at 12 weeks post-treatment (SVR_12_) and the G/P pharmacokinetics by cirrhosis status.

### Statistical Analysis

Analyses of safety, efficacy, and pharmacokinetic data were performed using the integrated analysis set in the intent-to-treat (ITT) population. Statistical comparisons between subpopulations, based on cirrhosis status, were conducted on baseline demographics and disease characteristics, excluding fibrosis stage, Child-Pugh score, and treatment duration, using a Chi-square test. The number and percentage of patients who received G/P with treatment-emergent AEs and laboratory abnormalities (grade ≥3 and higher than baseline) were summarized by cirrhosis status and the presence or absence of CKD stages 4 or 5. Exposures of glecaprevir and pibrentasvir were assessed by population pharmacokinetic analyses using a nonlinear, mixed-effects modeling approach in NONMEM 7.3 [20]. The number and percentage of patients in the ITT population achieving SVR_12_ by cirrhosis status were summarized, with 2-sided 95% confidence intervals (CIs) calculated using the Wilson score method. A further analysis of SVR_12_ utilized a modified ITT population that excluded subjects who did not achieve SVR_12_ for reasons other than virologic failure (eg, patients who discontinued early or were lost to follow-up).

### Role of the Funding Source

AbbVie sponsored the studies (NCT02243280, NCT02243293, NCT02604017, NCT02640482, NCT02640157, NCT02636595, NCT02642432, NCT02651194, and NCT02446717), contributed to their design, the collection, and the analyses (including the current integrated analysis) and interpretation of the data, and participated in the writing, review, and approval of the manuscript. AbbVie is committed to responsible data sharing regarding the clinical trials we sponsor. This includes access to anonymized, individual, and trial-level data (analysis data sets), as well as other information (eg, protocols and Clinical Study Reports), as long as the trials are not part of an ongoing or planned regulatory submission. This includes requests for clinical trial data for unlicensed products and indications.

## RESULTS

### Baseline Patient Demographics and Disease Characteristics

This analysis included 2369 patients with chronic HCV genotypes 1–6 and with compensated liver disease who were enrolled in Phase II and Phase III clinical trials between 7 October 2014 and 13 May 2016. In total, 308 (13%) patients with compensated cirrhosis were enrolled (Table 1). Overall, the majority of patients were white, male, and HCV treatment-naive. Compared to patients without cirrhosis, patients with compensated cirrhosis more frequently were ≥65 years old (13% vs 21%), had a body mass index ≥30 kg/m^2^ (19% vs 37%), had prior HCV treatment experience (29% vs 41%), had HCV genotype 3 (26% vs 38%), and had a medical history of diabetes (7% vs 20%) and cardiovascular disease (30% vs 50%), whereas a lower percentage had HCV genotype 2 (21% vs 12%). Patients with compensated cirrhosis primarily had baseline Child-Pugh scores of 5 (86%), with >100 × 10^9^ platelets/L (77%) and >3.5 g/dL albumin (93%). Patients with CKD stages 4 or 5 from EXPEDTION-4 (n = 104) were also included, comprising 7% (20/272) of patients with compensated cirrhosis and 4% (84/1966) of patients without cirrhosis (Table 1).

**Table 1.** Baseline Demographics and Disease Characteristics

Characteristic, n (%)	Compensated Cirrhosis	Without Cirrhosis	Overall Population
	(n = 308)	(n = 2061)	(N = 2369)
Male^a^	199 (65)	1119 (54)	1318 (56)
Age ≥65 years^a^	64 (21)	264 (13)	328 (14)
BMI ≥30 kg/m^2a^	115 (37)	387 (19)	502 (21)
Race^a^			
White^a^	261 (85)	1637 (80)	1898 (80)
Black or African American	25 (8)	124 (6)	149 (6)
Asian^a^	17 (6)	255 (12)	272 (11)
Other	5 (2)	42 (2)	47 (2)
Missing	0	3	3
HCV RNA ≥1 000 000 IU/mL	183 (59)	1224 (59)	1407 (59)
HCV GT^a^			
GT1	123 (40)	864 (42)	987 (42)
GT2^a^	38 (12)	439 (21)	477 (20)
GT3^a^	116 (38)	527 (26)	643 (27)
GT4	22 (7)	160 (8)	182 (8)
GT5	2 (<1)	30 (1)	32 (1)
GT6	7 (2)	41 (2)	48 (2)
HCV treatment-experienced^a^	126 (41)	603 (29)	729 (31)
Type of prior HCV treatment^b^			
PRS-experienced	99 (79)	517 (86)	616 (84)
PI and/or NS5A inhibitor-based	27 (21)	86 (14)	113 (16)
Baseline fibrosis stage			
F0–F1	0	1651 (80)	1651 (70)
F2	2 (<1)^c^	163 (8)	165 (7)
F3	2 (<1)^c^	243 (12)	245 (10)
F4	303 (98)	0	303 (13)
Missing	1^c^	4	5
Baseline Child-Pugh Score			
5	264 (86)	4 (<1)	268 (11)
6	41 (13)	0	41 (2)
>6	2 (<1)	0	2 (<1)
Missing	1	2057	2058
Platelet count <100 × 10^9^ cells/L^a^	70 (23)	7 (<1)	77 (3)
Albumin <3.5 g/dL^a^	23 (7)	5 (<1)	28 (1)
CKD stages 4 or 5^a^ (eGFR <30 ml/min/1.73 m^2^)	20 (7)	83 (4)	103 (5)
Missing CKD stage	36	95	131
History of diabetes^a^	63 (20)	141 (7)	204 (9)
History of cardiovascular disease^a^	154 (50)	622 (30)	776 (33)
G/P treatment duration			
8 weeks	0	828 (40)	828 (35)
12 weeks	245 (80)	1176 (57)	1421 (60)
16 weeks	63 (20)	57 (3)	120 (5)

Percentages were calculated on nonmissing values. Chi-square tests were conducted on characteristics, excluding fibrosis stage, Child-Pugh Score, and treatment duration.

Abbreviations: BMI, body mass index; CKD, chronic kidney disease; eGFR, estimated glomerular filtration rate; G/P, glecaprevir/pibrentasvir; GT, genotype; HCV, hepatitis C virus; IFN, interferon; PI, protease inhibitor; PRS, experienced with IFN, pegylated IFN ± ribavirin, or sofosbuvir + ribavirin.

^a^Statistically significant different between patients with compensated cirrhosis and patients without cirrhosis at 0.05 level. Since there are statistically significant differences in the distribution of race and HCV GT, each race and each HCV GT were tested using a chi-square test for 2 x 2 contingency tables, except for HCV GT 5, where some of the expected cell counts were less than 5.

^b^Percentages were calculated out of the total number of treatment-experienced patients.

^c^Patients who were classified as cirrhotic by the investigator during screening had postbaseline Fibroscan results of fibrosis stage F4, confirming their cirrhotic status.

### Safety Outcomes

Among patients without CKD stages 4 or 5, the rates of AEs were 74% (213/288) and 67% (1316/1977) in patients with compensated cirrhosis and without cirrhosis, respectively. The majority of patients had AEs with a maximum severity of mild (Grade 1), regardless of cirrhosis status (Table 2). Headache and fatigue were the most commonly reported AEs, occurring at similar frequencies irrespective of the presence of cirrhosis. There were 3 patients without cirrhosis who experienced a total of 9 DAA-related AEs that led to study drug discontinuation, including abdominal pain, diarrhea, nausea, fatigue, malaise, dizziness, headache, and transient ischemic attack. Only 1 (<0.1%) patient without cirrhosis experienced a serious AE (SAE) that was assessed by the investigator as related to G/P. This patient had a history of smoking, obesity, and a cardiac conduction abnormality, along with elevated hemoglobin and hematocrit at screening and, on Day 11, the patient had an SAE of a transient ischemic attack, leading to treatment discontinuation on Day 12 and the subsequent resolution of the SAE on the same day. This patient subsequently experienced another SAE of transient ischemic attack on Day 36 (24 days after discontinuing G/P treatment).

**Table 2.** Treatment-emergent Adverse Events

Event, n (%)	Without CKD Stages 4 or 5			With CKD Stages 4 or 5			Overall
	Compensated Cirrhosis	Without Cirrhosis	Total	Compensated Cirrhosis	Without Cirrhosis	Total	
	(n = 288)	(n = 1977)	(n = 2265)	(n = 20)	(n = 84)	(n = 104)	(N = 2369)
Any AE	213 (74)	1316 (67)	1529 (68)	20 (100)	54 (64)	74 (71)	1603 (68)
Any grade ≥3 AE	20 (7)	45 (2)	65 (3)	11 (55)	14 (17)	25 (24)	90 (4)
Serious AE	17 (6)	31 (2)	48 (2)	11 (55)	14 (17)	25 (24)	73 (3)
DAA-related^a^ serious AE	0	1 (<1)	1 (<1)	0	0	0	1 (<1)
AE leading to study drug discontinuation	0	8 (<1)^b^	8 (<1)^b^	2 (10)^c^	2 (2)	4 (4)^c^	12 (<1)
AEs in ≥10% of patients^d^							
Headache	47 (16)	363 (18)	410 (18)	1 (5)	8 (10)	9 (9)	419 (18)
Fatigue	58 (20)	272 (14)	330 (15)	1 (5)	14 (17)	15 (14)	345 (15)
Nausea	27 (9)	181 (9)	208 (9)	4 (20)	8 (10)	12 (12)	220 (9)
Pruritus	18 (6)	85 (4)	103 (5)	6 (30)	15 (18)	21 (20)	124 (5)
Any HCC^e^	5 (2)	1 (<1)	6 (<1)	0	0	0	6 (<1)
Treatment-emergent HCC	3 (1)	0	3 (<1)	0	0	0	3 (<1)
AEs consistent with hepatic decompensation	1 (<1)	0	1 (<1)	0	0	0	1 (<1)
Deaths	1 (<1)^f^	5 (<1)^g^	6 (<1)	1 (5)^f^	0	1 (<1)	7 (<1)

Abbreviations: AE, adverse event; CKD, chronic kidney disease; DAA, direct-acting antiviral; HCC, hepatocellular carcinoma.

^a^Relatedness of AEs to DAA treatment was determined by a study investigator.

^b^Of these 8 patients, 3 experienced a total of 9 DAA-related AEs that led to study drug discontinuation, including abdominal pain, diarrhea, nausea, fatigue, malaise, dizziness, headache, and transient ischemic attacks.

^c^Of these 4 patients, 2 with compensated cirrhosis experienced a DAA-related AE, 1 experienced Grade 2 diarrhea, and the other experienced Grade 3, worsening pruritus.

^d^AEs occurring in ≥10% of all patients with CKD stage 4 or 5, or all patients without CKD stage 4 or 5.

^e^Includes 3 events of HCC that occurred after 30 days post-treatment and, thus, were not classified as treatment-emergent events of HCC.

^f^Cause of death was cerebral hemorrhage in both patients with compensated cirrhosis.

^g^The causes of death for these patients were pneumonia, accidental overdose, adenocarcinoma, hepatic cancer metastatic, and acute ethanol and combined methadone toxicity.

As expected due to underlying renal disease and associated comorbidities [21, 22], the 104 patients with CKD stages 4 or 5, 82% of whom were on dialysis, had greater rates of SAEs; however, most SAEs were considered unrelated to DAA treatment (Table 2). Pruritus, fatigue, and nausea were the most commonly reported AEs, all of which most commonly occurred in patients on dialysis. Pruritus is common amongst patients with CKD on dialysis, including those with a chronic HCV infection [23]. There were 2 patients with both cirrhosis and CKD stage 4 or 5 who experienced nonserious AEs of diarrhea and worsening pruritus, respectively, which led to the premature discontinuation of G/P.

Overall, AEs consistent with hepatic decompensation and treatment-emergent HCC were rare, occurring in 1 and 6 of 2369 patients (<1%), respectively (Table 2). Among those with compensated cirrhosis, 1 (<1%) patient with a history of esophageal varices had variceal bleeding on Day 22, which was considered not related to G/P; the patient did not discontinue G/P and there was no evidence of a deteriorating hepatic function or concurrent change in ALT or total bilirubin. De novo HCC was reported in 6 patients (5 with compensated cirrhosis), of which 3 cases were classified as treatment-emergent based on criteria described in the Methods. None were considered related to G/P, but were considered related to underlying cirrhosis or long-standing chronic HCV infection (see Supplementary Table 2 for more information). Of these 6 patients with de novo HCC, 5 achieved SVR_12_, while 1 patient died prior to post-treatment Week 12 from metastatic hepatic cancer. All 7 deaths were considered not related to G/P (see Table 2 and Supplementary Table 3 for more information).

 There were no ALT elevations consistent with hepatotoxicity. ALT elevations (Grade ≥3) were observed in 2 of 2369 (<1%) patients, neither of whom had cirrhosis (Table 3). In 1 of these 2 patients, the ALT elevation was in the context of multiple gallstones. For the second patient, the Grade 3 ALT was observed on Day 7 of treatment, after Grade 2 ALT on Day 3 and from Grade 3 ALT at baseline, which is attributed to common ALT fluctuation during the first week of therapy. No patients discontinued or interrupted therapy due to ALT elevations.

**Table 3.** Laboratory Abnormalities

Grade ≥3,^a^ n (%)	Compensated Cirrhosis	Without Cirrhosis	Overall Population
	(n = 308)	(n = 2061)	(N = 2369)
ALT >5 × ULN^b^	0	2 (<1)	2 (<1)
AST >5 × ULN	0	6 (<1)	6 (<1)
Total bilirubin >3 × ULN	3 (1)^c^	6 (<1)	9 (<1)
Platelets <50 × 10^9^/L	4 (1)	0	4 (<1)

Abbreviations: ALT, alanine aminotransferase; AST, aspartate aminotransferase; CKD, chronic kidney disease; ULN, upper limit of normal.

^**a**^Grade ≥3 lab abnormality in the specific parameter tested that is more extreme than the baseline grade.

^b^Postnadir increase in grade to Grade ≥3.

^c^Includes 1 patient with CKD stages 4 or 5 who experienced a Grade 3 elevation in total bilirubin.

Grade 3 (>3 × upper limit of normal) elevations in total bilirubin occurred in approximately 1% of patients, regardless of cirrhosis status, and most of these patients had preexisting indirect bilirubin elevations (Grade 1 or 2). Most Grade 3 elevations in total bilirubin were transient in nature and predominantly resulted from increased, indirect bilirubin fractions, which is consistent with the known mechanism that glecaprevir can impact bilirubin transport and conjugation [10].

### Efficacy

**Figure 1.**
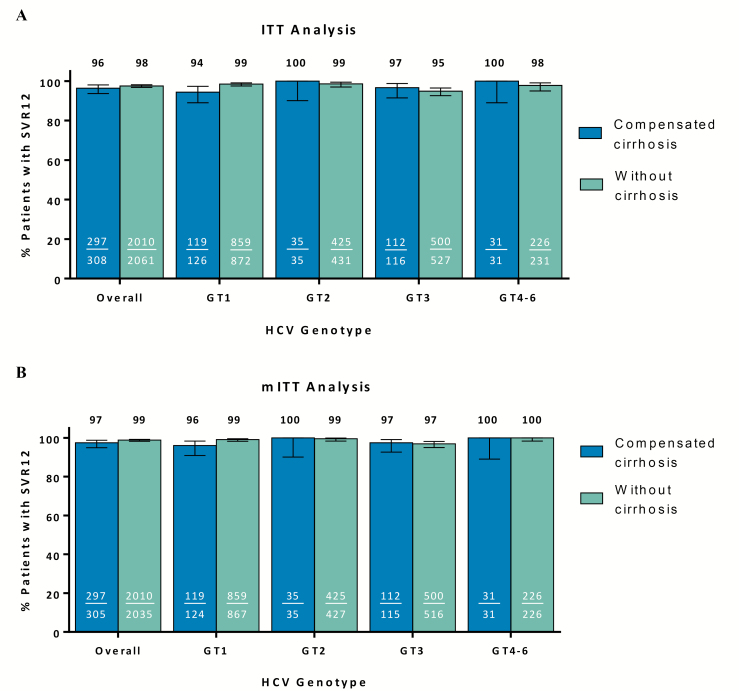
Sustained virologic response at 12 weeks post-treatment (SVR_12_) by cirrhosis status and HCV genotype. Glecaprevir/pibrentasvir efficacy, defined as SVR_12_, reported by cirrhosis status and further stratified by HCV GT using ITT (*A*) and modified ITT (*B*) analyses. Abbreviations: GT, genotype; HCV, hepatitis C virus; ITT, intent-to-treat.

Overall, SVR_12_ rates in the ITT population were 96.4% (297/308; 95% CI 93.7–98.0%) and 97.5% (2010/2061; 95% CI 96.8–98.1%) in patients with compensated cirrhosis and without cirrhosis, respectively (Table 5). Rates of not achieving SVR_12_ were similar between patients with compensated cirrhosis and without cirrhosis. There were 5 (1.6%) patients with cirrhosis who experienced on-treatment virologic failure, 2 of whom had prior treatment experience with both an NS5A inhibitor and a NS3/4A protease inhibitor. SVR_12_ rates, stratified by genotype, are reported in Figure 1 for the ITT and modified ITT populations.

**Table 5.** Summary of Intent-to-treat Efficacy Outcomes

Outcome	Compensated Cirrhosis	Without Cirrhosis
	(n = 308)	(n = 2061)
SVR_12_, % (n/N) [95% CI]	96.4 (297/308) [93.7–98.0]	97.5 (2010/2061) [96.8–98.1]
**Reason for nonresponse, n (%)**		
On-treatment virologic failure	5^a^	6
Relapse	3	19
Premature study drug discontinuation	1	11
Missing SVR_12_ data	2	15

Abbreviations: CI, confidence interval; DAA, direct-acting antiviral; SVR_12_, sustained virologic response at 12 weeks post-treatment.

^a^There were 2 patients who had prior treatment experience with both a NS5A inhibitor and NS3/4A protease inhibitor. Glecaprevir/pibrentasvir are not recommended for treatment in this dual DAA-experienced patient population.

### Pharmacokinetics

Steady-state exposures of glecaprevir and pibrentasvir are reported in Table 4 by cirrhosis status and expressed as the area under the curve (ng*hr/mL). Mean glecaprevir exposure was 2.2-fold higher in patients with compensated cirrhosis (10900 ng*hr/mL), compared to those without cirrhosis (4940 ng*hr/mL). The pibrentasvir mean exposure was similar in patients with compensated cirrhosis (1560 ng*hr/mL), compared to patients without cirrhosis (1460 ng*hr/mL). The effect of renal impairment on G/P exposure was similar to those effects observed previously, and was not considered clinically significant [10, 11, 24].

**Table 4.** Geometric Mean and Variability of Steady-state Pharmacokinetic Data by Cirrhosis Status

AUC_24_, ng^a^hr/mL (% CV)	Compensated Cirrhosis	Without Cirrhosis
	(n = 308^a^)	(n = 2056^a,b^)
Glecaprevir	10 900 (106)	4940 (124)
Pibrentasvir	1560 (55)	1460 (57)

Abbreviations: AUC_24_, area under the concentration-time curve; CKD, chronic kidney disease; CV, coefficient of variation.

^a^Includes all patients with CKD stages 4 or 5.

^b^N derived from patients without cirrhosis with available data in Phase 2 and 3 studies. Pharmacokinetic data were missing for 5 patients without cirrhosis who were enrolled in the dedicated, genotype 3–infected clinical trial, ENDURANCE-3.

## DISCUSSION

Overall, our analysis shows low rates (≤2%) of ALT or bilirubin elevations, of AEs leading to the discontinuation of G/P, and of G/P-related SAEs in 2369 HCV-infected patients treated with G/P in the 9 Phase II and III G/P clinical trials. Despite the increased exposure of glecaprevir in patients with compensated cirrhosis (n = 308) enrolled across the G/P clinical trials, these patients reported AEs that were similar in type, frequency, and severity to those experienced by patients without cirrhosis. No cases consistent with a drug-induced liver injury were reported. This is consistent with the findings from the dedicated Phase III trial for patients with compensated cirrhosis, EXPEDITION-1 (n = 146), extending its findings to demonstrate the favorable safety profile of G/P in a larger cohort of patients with compensated cirrhosis [8].

Based on exposure-safety analyses for over 2600 patients in Phase II and Phase III studies who received glecaprevir and pibrentasvir, patients with renal impairment and/or compensated cirrhosis had similar safety profiles, even with higher glecaprevir exposures. No exposure–response relationships were identified between glecaprevir or pibrentasvir exposures and ALT elevations or other AEs [8, 25].

The Phase III trial (EXPEDITION-4) was conducted in patients with CKD stages 4 or 5, in part to characterize the unique safety profile in the CKD population, in whom reported AEs are unrelated to HCV chronic infection or DAA treatment [21, 22]. There were no clinically significant changes in G/P exposures of patients with any degree of renal impairment, including dialysis, and, thus, G/P is recommended without dose reduction in patients with CKD stage 4 or 5, including those on dialysis [24]. In EXPEDITION-4, there were no safety concerns due to G/P in patients with CKD stages 4 or 5 (n = 104, including 85 on hemodialysis), including in 20 (19%) patients with compensated cirrhosis [19]. Common AEs and SAEs were reported more frequently amongst patients with CKD, and these were primarily attributed to underlying renal disease and associated comorbidities [21–23]. Thus, through the inclusion of these patients with CKD stages 4 or 5, this integrated analysis highlights the favorable safety profile of G/P, supporting its use in patients with compensated liver disease, whether with or without renal impairment, including patients on chronic renal dialysis.

Limitations of our analysis include the lack of active or placebo controls for trials evaluating HCV-infected patients with cirrhosis and/or CKD stages 4 or 5. However, the large number of patients in the analysis allows for a robust comparison of the safety of G/P in cirrhotic compared to noncirrhotic patients, including subpopulations such as patients with CKD stage 4 or 5. Additionally, as a post hoc analysis, none of the analyses in this integrated manuscript were prespecified. This integrated analysis did not include patients with a liver or renal transplant. Phase IIIb clinical trials previously reported the favorable safety profile of G/P in patients with liver or renal transplants [26].

Overall, this integrated analysis shows that the all-oral, once-daily regimen of glecaprevir coformulated with pibrentasvir is a safe and effective treatment option for patients with compensated liver disease and/or with any degree of renal impairment, including patients on dialysis. As a RBV-free and pangenotypic regimen, G/P offers healthcare providers a simple treatment algorithm across all HCV genotypes, including in patients with compensated liver disease with or without renal impairment.

## Supplementary Data

Supplementary materials are available at *Clinical Infectious Diseases* online. Consisting of data provided by the authors to benefit the reader, the posted materials are not copyedited and are the sole responsibility of the authors, so questions or comments should be addressed to the corresponding author.

ciz022_suppl_Supplementary_Material

## Notes

***Author contributions.*** E. G., N. Z., C. Setze, Y. Y., and F. J. M. designed the study. C. Setze, Y. Y., and T. P.-M. led the study analyses. All authors contributed to the implementation, conduct, data interpretation, writing, and review of this work. All authors approved the final version of the manuscript and the decision to submit to the journal.

***Acknowledgments.*** The authors thank the study participants, researchers, and staff for their contributions to the studies included in this integrated analysis. They acknowledge Dan O’Brien, of AbbVie, for providing medical writing support for this manuscript.

***Financial support.*** This work was supported by AbbVie, Inc., North Chicago, Illinois, including the integrated analysis and all studies included in the analysis.

***Potential conflicts of interest.*** AbbVie sponsored the studies, contributed to their designs, the data collection, the analyses, the integrated analysis, and the interpretation of the data, and participated in the writing, review, and approval of the manuscript. E. G. is an advisor for AbbVie, Alnylam, Arbutus, Assembly, Arrowhead, Enanta, Gilead Sciences, Janssen, Merck, Novartis, Novira, Roche, and VIR and participated in speakers’ bureaus for AbbVie and Gilead Sciences. F. P. received research support from AbbVie, Bristol-Myers Squibb, Gilead Sciences, Intercept Pharmaceuticals, and Merck; participated as a speaker for AbbVie, Bristol-Myers Squibb, Gilead, Intercept Pharmaceuticals, Merck, and Salix; and served as a consultant/advisor for AbbVie, Bristol-Myers Squibb, Gilead Sciences, and Merck. C. S. served as a consultant/advisor for Janssen, AbbVie, Gilead, and Merck Sharp & Dohme Corp. and participated as a speaker for AbbVie and Gilead Sciences. S. G. was a participant in AbbVie-sponsored clinical trials.

T. N. received research support from Gilead, Bristol-Myers Squibb, and Idenix Pharmaceuticals and served as an advisor/speaker for Gilead and Bristol-Myers Squibb. M. E. was a participant in AbbVie-sponsored clinical trials. M.-A. W. served as an advisor for AbbVie and Bristol-Myers Squibb and participated as a speaker for AbbVie, Bristol-Myers Squibb, Gilead, Merck Sharp & Dohme Corp., and Janssen. A. T. participated as a speaker and investigator for AbbVie, Gilead Sciences, and Merck Sharp & Dohme Corp. J.-P. M. received research grants from Janssen and Gilead; served as an advisor for Janssen, Gilead, Merck Sharp & Dohme Corp., Bristol-Myers Squibb, and AbbVie; and participated as a speaker for Bristol-Myers Squibb and Gilead. N. Z., J. V., C.-W. L., W. L., A. A., S. W., Y. Y., C. S., T. P.-M., A. P., and F. J. M. are employees of AbbVie and may hold stock or options. All authors have submitted the ICMJE Form for Disclosure of Potential Conflicts of Interest. Conflicts that the editors consider relevant to the content of the manuscript have been disclosed.
