# Supplementary material for: Safety and Pharmacokinetics of Glecaprevir/Pibrentasvir in Adults With Chronic Genotype 1–6 Hepatitis C Virus Infections and Compensated Liver Disease
Source: Clin Infect Dis. 2019 Mar 28;69(10):1657–64. doi: 10.1093/cid/ciz022 (PMC6821220; doi:10.1093/cid/ciz022)
Supplement: ciz022_suppl_Supplementary_Material [file ciz022_suppl_supplementary_material.docx]

# Online Supporting Information

## Safety and Pharmacokinetics of Glecaprevir/Pibrentasvir in Adults With Chronic Genotype 1-6 Hepatitis C Virus Infection and Compensated Cirrhosis

## Edward Gane^1^, Fred Poordad^2^, Neddie Zadeikis^3^, Joaquin Valdes^3^, Chih-Wei Lin^3^, Wei Liu^3^, Armen Asatryan^3^, Stanley Wang^3^, Catherine Stedman^4^, Susan Greenbloom^5^, Tuan Nguyen^6^, Magdy Elkhashab^7^, Marcus-Alexander Wörns^8^, Albert Tran^9^, Jean-Pierre Mulkay^10^, Yao Yu^3^, Tami Pilot-Matias^3^, Ariel Porcalla^3^, Federico J Mensa^3^

## Table of Contents

HCV RNA Assays…………………………………………………………………………………………………………………………………..2

[Eligibility Criteria 3](#_Toc479596269)

[Inclusion 3](#_Toc479596270)

[Exclusion 8](#_Toc479596270)

[Supporting Figure 1. Phase 2 and 3 Clinical Trials for G/P. 11](#_Toc479596272)

Supporting Figure 2. Patient disposition. 12

Supporting Table 1. Medications Contraindicated for Use with Study Drug Regimen…………….………13

Supporting Table 2. Cases of treatment-emergent and non-treatment emergent HCC………………….14

[Supporting Table 3. Deaths for G/P-treated Patients by Cirrhosis Status.. 15](#_Toc479596273)

## HCV RNA Assays

For the 201 patients enrolled in Phase 2 trials as well as 203 patients enrolled in the Phase 3 SURVEYOR-II Part 4 study, specimen preparation was done manually with the High Pure System and plasma HCV RNA levels were determined for each sample collected by the central laboratory using the COBAS TaqMan® real-time reverse transcriptase-polymerase chain reaction (RT-PCR) assay v. 2.0 (Roche Molecular Diagnostics, Pleasanton, CA), which has a LLOQ of 25 IU/mL, regardless of genotype. The LLOD is 15.0 for genotypes 1 and 3, and 5.6, 12.0, 3.7, and 20.4 IU/mL for HCV genotype 2, 4, 5, and 6, respectively. For patients enrolled in Phase 3 trials (N=1965, excluding the 203 enrolled in SURVEYOR-II Part 4), plasma HCV RNA levels were determined for each sample collected by the central laboratory using the COBAS Ampliprep/TaqMan® real-time RT-PCR assay v. 2.0 (Roche Molecular Diagnostics, Pleasanton, CA), which has a lower limit of quantification (LLOQ) and a lower limit of detection (LLOD) of 15 IU/mL, regardless of genotype.

## Eligibility Criteria

### Inclusion

### Male or female, at least 18 years of age at time of Screening with an upper limit of 70 years of age only in Phase 2 trials.

### If female, subject must be either:

### Practicing one effective method of birth control with male partner(s) from screening to 30 days after stopping study drug

### Postmenopausal for at least 2 years prior to screening

### Or permanently surgically sterile (defined as bilateral tubal ligation, bilateral oophorectomy, or hysterectomy) or has a vasectomized partner(s)

### Females of childbearing potential must have a negative serum pregnancy test result at Screening, and a negative urine pregnancy test at Study Day 1.

### Females of non-childbearing potential (either postmenopausal or permanently surgically sterile) at Screening do not require pregnancy testing.

### Sexually active males must be surgically sterile or have male partners only, or if sexually active with female partner(s) of childbearing potential must agree to practice at least one effective form of birth control

### Screening laboratory result indicating HCV GT1-, 2-, 3-, 4-, 5-, or 6-infection.

### Subject has positive anti-HCV Ab and plasma HCV RNA viral load ≥10000 IU/mL in Phase 2 trials and ≥1000 IU/mL in Phase 3 trials at Screening Visit.

### Chronic HCV infection defined as one of the following:

### Positive for anti-HCV antibody (Ab) or HCV RNA at least 6 months before Screening; or

### A liver biopsy consistent with chronic HCV infection; or

### Abnormal alanine aminotransferase (ALT) levels for at least 6 months before Screening (only used in Phase 3 trials).

### Subject must be HCV treatment-naïve (i.e., subject has not received a single dose of any approved or investigational anti-HCV medication) or HCV treatment-experienced (subject has failed prior IFN or pegIFN with or without RBV or SOF plus RBV with or without pegIFN). GT3 subjects must be HCV treatment-naïve. Previous HCV treatment must have been completed ≥ 1 month prior to Screening.

1. Body Mass Index (BMI) is ≥ 18.0 kg/m^2^ at the time of Screening with an upper limit of 38.0 kg/m with an upper limit of 38.0 kg/m^2^ only in Phase 2 trials. BMI is calculated as weight measured in kilograms (kg) divided by the square of height measured in meters (m).
2. Subject must be documented as non-cirrhotic or cirrhotic defined as meeting one of the following criteria:

**Non-Cirrhotics**

- - A liver biopsy within 24 months prior to or during Screening demonstrating the absence of cirrhosis, e.g., a METAVIR, Batts-Ludwig, Knodell, IASL, Scheuer, or Laennec fibrosis score of ≤ 3, Ishak fibrosis score of ≤ 4; or
  - A FibroScan® score of < 12.5 kPa within ≤ 6 months of Screening or during Screening period (FibroScan® must be approved by the local regulatory agency to qualify for entrance criteria); or
    1. Subjects with indeterminate FibroScan® score (12.5≤ score < 14.6), must have a qualifying liver biopsy
  - A Screening FibroTest score of ≤ 0.48 and Aspartate Aminotransferase to Platelet Ratio Index (APRI) < 1.
    1. Subjects with indeterminate Fibrotest (0.48< result <0.75), or conflicting FibroTest and APRI results (e.g., FibroTest ≤ 0.48, but APRI ≥ 1) must have a qualifying liver FibroScan® or biopsy.

1. Subject must voluntarily sign and date an informed consent form, approved by an Institutional Review Board (IRB)/Independent Ethics Committee (IEC) prior to the initiation of any Screening or study specific procedures.
2. Subjects must be able to understand and adhere to the study visit schedule and all other protocol requirements.

**For MAGELLAN-I, SURVEYOR-I and –II, and EXPEDITION-1 and -4**

**Cirrhotic**

- - Previous histologic diagnosis of cirrhosis on liver biopsy, e.g., METAVIR, Batts-Ludwig, Knodell, IASL, Scheuer, or Laennec fibrosis score of > 3, Ishak score of > 4 or on a liver biopsy conducted during Screening; or
  - A FibroScan® score of < 12.5 kPa within ≤ 6 months of Screening or during Screening period (FibroScan® must be approved by the local regulatory agency to qualify for entrance criteria); or
  - A Screening FibroTest result that is ≥ 0.75 and an APRI > 2.

In the absence of a definitive diagnosis of presence or absence of cirrhosis by Fibrotest/APRI using the above criteria (indeterminate FibroTest [0.48 < result < 0.75], or conflicting FibroTest and APRI results [e.g., FibroTest ≤ 0.48, but APRI ≥ 1]), a liver biopsy or FibroScan® is required. Liver biopsy results will supersede Fibrotest/APRI or FibroScan® results and be considered definitive.

FibroScan® results will supersede Fibrotest/APRI results for the determination of presence or absence of cirrhosis

1. Cirrhotic Subjects Only: Compensated cirrhosis defined as Child-Pugh score of ≤ 6 at Screening and no current or past evidence of Child-Pugh B or C Classification or clinical history of liver decompensation including ascites noted on physical exam, hepatic encephalopathy or esophageal variceal bleeding.
2. Cirrhotic Subjects Only: Absence of hepatocellular carcinoma (HCC) as indicated by a negative ultrasound, computed tomography (CT) scan or magnetic resonance imaging (MRI) within 3 months prior to Screening or a negative ultrasound at Screening. Subjects who have an ultrasound with results suspicious of HCC followed by a subsequent negative CT or MRI of the liver will be eligible for the study.

**For MAGELLAN-1 only**

1. History of previous direct-acting antiviral-containing treatment (which was either approved at the time of treatment, or if investigational, then approval of AbbVie must have been obtained; examples of investigational therapies in Part 1 include, but are not limited to, DCV + SMV, DCV + SOF, ASV + DCV, SOF + SMV, OBV + PTV/r for chronic HCV genotype 1 infection and in Part 2 were limited to combination regimens (with or without IFN and/or RBV) consisting of NS5A-inhibitors DCV, LDV, or OBV, NS3/4A PIs PTV/r, SMV, TVR, or BOC, with treatment outcome as either on-treatment virologic failure or
2. Post-treatment relapse, defined as:
   - On-Treatment Failure: The patient will be considered to have experienced on-treatment failure of the prior direct-acting antiviral-containing treatment regimen if a) the patient did not achieve unquantifiable HCV RNA prior to or by the planned end of the direct-acting antiviral-containing therapy (including those with on-treatment virologic breakthrough after achieving unquantifiable HCV RNA), or if b) the patient was documented to have met futility criteria as defined in the product label (e.g., for TVR or BOC containing regimens); or
   - Post-Treatment Relapse: The patient will be considered to have experienced post-treatment relapse if the HCV RNA was < LLOQ at the planned end of the prior direct-acting antiviral-containing treatment regimen, but was confirmed to be quantifiable after the end-of-treatment
3. Treatment must have been completed at least 1 month prior to Screening Visit

**For ENDURANCE-1 only**

1. Positive test result for anti-Human Immunodeficiency Virus antibody at Screening.
2. Naïve to treatment with any antiretroviral therapy (ART) (and have no plans to initiate ART treatment while participating in this study), or

On a stable, qualifying HIV-1 ART regimen for at least 8 weeks prior to Screening. The HIV-1 ART regimen must include at least one of the following ARV agents:

- - For cirrhotic and non-cirrhotic subjects:
    1. Raltegravir (RAL) PO BID
    2. Dolutegravir (DTG) PO QD or PO BID
    3. Rilpivirine (RPV) PO QD
    4. Elvitegravir/cobicistat (EVG/COBI) PO QD
  - For non-cirrhotic subjects, the following regimens are also allowed:
    1. Darunavir (DRV) co-administered with ritonavir (RTV) PO QD
    2. Darunavir/cobicistat (DRV/COBI) PO QD
    3. Lopinavir/ritonavir (LPV/r) PO BID

In addition to the above medications, subjects (both cirrhotic and non-cirrhotic) may take a nucleoside/nucleotide reverse transcriptase inhibitor (N(t)RTI) backbone containing any of the following:

- - Tenofovir disoproxil fumarate (TDF) PO QD
  - Tenofovir alafenamide (TAF) PO QD
  - Abacavir (ABC) PO QD or BID
  - Emtricitabine (FTC) PO QD
  - Lamivudine (3TC) PO QD or BID

Subjects receiving any other HIV-1 ART in addition to those noted above would not be eligible for enrollment in the study.

1. Subjects naïve to ART must have the following:
   - CD4+ count ≥ 500 cells/mm3 (or CD4+ % ≥ 29%) at Screening; and
   - Plasma HIV-1 RNA < 1,000 copies/mL at Screening (by the COBAS® Ampliprep/COBAS® Taqman HIV-1 Test, v 2.0) and at least once during the 12 months prior to Screening (by an approved plasma HIV-1 RNA quantitative assay including but not limited to: COBAS® Ampliprep/COBAS® Taqman HIV-1 Test, v 2.0 or Abbott RealTime HIV-1 assay).
2. Subjects on a stable ART regimen must have the following:
   - CD4+ count ≥ 200 cells/mm3 (or CD4+ % ≥14%) at Screening; and
   - Plasma HIV-1 RNA below LLOQ at Screening (by the COBAS® Ampliprep/COBAS® Taqman HIV-1 Test, v 2.0) and at least once during the 12 months prior to Screening (by an approved plasma HIV-1 RNA quantitative assay including but not limited to: COBAS® Ampliprep/COBAS® Taqman HIV-1 Test, v 2.0 or Abbott RealTime HIV-1 assay).

### Exclusion

1. Female subject who is pregnant, breastfeeding or is considering becoming pregnant during the study; or a male whose partner is pregnant or planning to become pregnant during the study.
2. Recent (within 6 months prior to study drug administration) history of drug or alcohol abuse that could preclude adherence to the protocol in the opinion of the investigator.
3. Subjects on peritoneal dialysis.
4. Positive test result at Screening for hepatitis B surface antigen (HBsAg; for all HCV genotypes 1-6) or Human Immunodeficiency virus (HIV) Ab (for HCV genotypes 2-6).
5. HCV genotype performed during Screening indicating co-infection with more than one HCV genotype.
6. Requirement for and inability to safely discontinue the medications or supplements listed in **Table 1** at least 2 weeks or 10 half-lives (whichever is longer) prior to the first dose of any study drug.
7. Clinically significant abnormalities or co-morbidities, other than HCV/HIV-1 co-infection, based upon the results of a medical history, physical examination, vital signs, laboratory profile, and a 12-lead electrocardiogram (ECG) that make the subject an unsuitable candidate for this study in the opinion of the investigator, including, but not limited to::

- Uncontrolled diabetes as defined by a glycated hemoglobin (hemoglobin A1C) level > 8.5% during Screening.
- Active or suspected malignancy or history of malignancy (other than basal cell skin cancer or cervical carcinoma in situ) in the past 5 years.
- Uncontrolled cardiac, respiratory, gastrointestinal, hematologic, neurologic, psychiatric, or other medical disease or disorder, which is unrelated to the existing HCV infection..

1. Any cause of liver disease other than chronic HCV-infection, including but not limited to the following:
   - Hemochromatosis.
   - Alpha-1 antitrypsin deficiency.
   - Wilson's disease.
   - Autoimmune hepatitis.
   - Alcoholic liver disease.
   - Steatohepatitis on liver biopsy considered to be the primary cause of the liver disease rather than concomitant/incidental with HCV infection.
2. Screening laboratory analyses showing any of the following abnormal laboratory results:
   - ALT >5 x ULN in Phase 2 trials or >10 × ULN in Phase 3 trials
   - AST >5 x ULN in Phase 2 trials or >10 × ULN in Phase 3 trials
   - Calculated creatinine clearance (using Cockcroft-Gault method) of < 50 mL/min (for all studies other than EXPEDITION-4)
   - Direct bilirubin > ULN
   - Albumin < 2.8 g/dL for patients with cirrhosis and <LLN for patients without cirrhosis
   - International normalized ratio (INR) > 1.5 × ULN, unless subject has known hemophilia or is on a stable anticoagulant regimen affecting INR
   - Hemoglobin <LLN
   - Platelets < 60,000 cells per mm^3^ for subjects with cirrhosis; < 90,000 cells per mm^3^ for subjects without cirrhosis
3. History of solid organ transplantation.
4. Receipt of any investigational product within a time period equal to 10 half-lives of the product, if known, or a minimum of 6 weeks (whichever is longer) prior to study drug administration.
5. Any current or past clinical evidence of decompensated liver disease such as ascites noted on physical exam, use of beta-blockers for portal hypertension, hepatic encephalopathy or esophageal variceal bleeding.
6. Consideration by the investigator, for any reason, that the subject is an unsuitable candidate to receive ABT-493/ABT-530.
7. Requirement for chronic use of systemic immunosuppressants including, but not limited to, corticosteroids (prednisone equivalent of > 10 mg/day for > 2 weeks), azathioprine, or monoclonal antibodies (e.g., infliximab).
8. History of severe, life-threatening or other significant sensitivity to any excipients of the study drug.
9. For subjects with HIV co-infection, treatment for an AIDS-associated opportunistic infection (OI) (Appendix E) within 6 months of Screening.
10. Patients who cannot participate in the study per local law.

## **Supporting Figure 1. Phase 2 and 3 Clinical Trials for G/P in patients with HCV GT1-6 infection either without cirrhosis or with compensated cirrhosis**


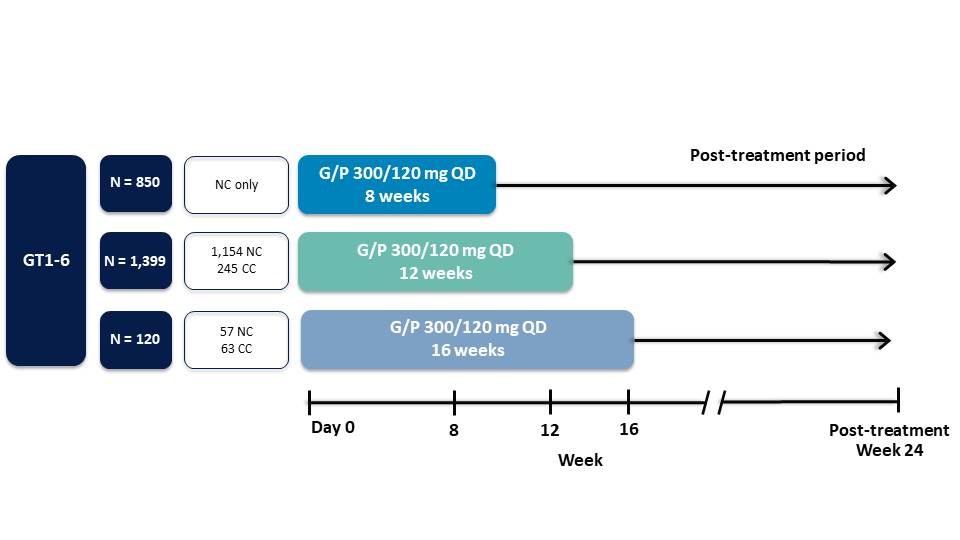


GT, genotype; NC, non-cirrhotic; CC, compensated cirrhosis; QD, once-daily

## **Supporting Figure 2.** Patient Disposition.

2376 patients randomized

7 patients not treated

998 treated patients with GT1 infection

13 discontinued treatment

4 due to AEs

998 analyzed for ITT

4 discontinued treatment

- 0 due to AEs

466 analyzed for ITT

14 discontinued treatment

- 3 due to AEs

643 analyzed for ITT

6 discontinued treatment

- 5 due to AEs

262 analyzed for ITT

262 treated patients with GT4-6 infection

643 treated patients with GT3 infection

466 treated patients with GT2 infection

## Supporting Table 1. Medications Contraindicated for Use with Study Drug

| **Prohibited Medications and Supplements** |
| --- |
| Any herbal supplement (including milk thistle), read yeast rice (monacolin K), St. John’s Wort |
| Carbamazepine, phenytoin, pentobarbital, phenobarbital, primidone, rifabutin, rifampin |
| Atorvastatin, lovastatin, simvastatin* |
| Astemizole, cisapride, terfenadine |

*Some HMG-CoA reductase inhibitors (including atorvastatin, lovastatin, or simvastatin) should not be taken with the study drugs. Subjects receiving these statins should either switch to pravastatin or rosuvastatin prior to the first dose of study drugs or may interrupt statin therapy throughout the treatment period and until 30 days after the last dose of study drug, based on investigator’s judgement. If switching to or continuing pravastatin or rosuvastatin, it is recommended to reduce the pravastatin dose by 50% or limit the rosuvastatin dose to 10 mg QD when taking with the study drugs.

## Supporting Table 2. Cases of treatment-emergent and non-treatment emergent HCC

| **Cirrhosis Status** | **Day of Onset*** | **Related to G/P?** | **Relevant Medical History** |
| --- | --- | --- | --- |
| Treatment-emergent HCC* | | | |
| Compensated cirrhosis | 85 | No | Cirrhosis, liver nodules |
| Compensated cirrhosis | 92 (8) | No | Cirrhosis |
| Compensated cirrhosis | 40 | No | Cirrhosis |
| Non-treatment emergent HCC† | | | |
| Noncirrhotic | 156 (71) | No | No Cirrhosis |
| Compensated cirrhosis | 122 (37) | No | Cirrhosis, benign hepatic nodules |
| Compensated cirrhosis | 189 (105) | No | Cirrhosis |
| HCC, hepatocellular carcinoma, G/P, glecaprevir/pibrentasvir  *Treatment-emergent HCC was defined as *de novo* HCC occurring after the first G/P dose and no more than 30 days after the last G/P dose  †Non-treatment emergent HCC was defined as *de novo* HCC occurring more than 30 days after the last G/P dose. There were no cases of HCC recurrence since patients with prior HCC were excluded from all G/P clinical trials. | | | |

## Supporting Table 3. Deaths of G/P-treated patients by Cirrhosis Status

| **Cirrhosis Status** | **Cause of Death** | **Day of Death*** | **Considered related to G/P?†** | **Relevant Medical History** | **Etiology(ies)** |
| --- | --- | --- | --- | --- | --- |
| MAGELLAN-I | | | | | |
| Noncirrhotic | Hepatic cancer metastatic | 156 (71) | No | None | Metastatic hepatocellular carcinoma with metastases to bone and lung |
| SURVEYOR-I |  |  |  |  |  |
| Noncirrhotic | Adenocarcinoma | 29 | No | Renal insufficiency and hypoechoic lymph nodes | Enlarged peripancreatic nodes |
| SURVEYOR-II | | | | | |
| Noncirrhotic | Pneumonia | 227 (141) | No | Chronic obstructive pulmonary disease, asthma, congestive heart failure | Chronic obstructive pulmonary disease |
| ENDURANCE-1 | | | | | |
| Noncirrhotic | Death | 99 (14) | No | Gastric bypass for obesity, heartburn, hypothyroidism, osteomyelitis, former injection drug abuser (on methadone for opioid substitution), and smoker | Acute ethanol and combined methadone toxicity |
| ENDURANCE-3 | | | | | |
| Noncirrhotic | Accidental overdose | 133 (77) | No | Suicidal ideation, anxiolytic and opioid dependence, intravenous drug use, opioid overdose | Pre-existing condition |
| EXPEDITION-1 | | | | | |
| Compensated cirrhosis | Cerebral hemorrhage | 143 (59) | No | von Willebrand disease Type III and previous bleeding episodes, including cerebral hemorrhages | Cerebral hemorrhage related to hemophilia |
| EXPEDITION-4 | | | | | |
| Compensated cirrhosis | Cerebral hemorrhage | 99 (14) | No | Hypertension, end stage renal disease, type 2 diabetes | Uncontrolled hypertension |
| *Numbers in parentheses indicate days after end of G/P treatment  †Determined by study investigator | | | | | |
